# Acute Coronary Artery Thrombosis in a Patient With Non-Small Cell Lung Cancer

**DOI:** 10.7759/cureus.12507

**Published:** 2021-01-05

**Authors:** Ismahane Lahmidi, Hanane Aissaoui, Nabila Ismaili, Noha Elouafi

**Affiliations:** 1 Cardiology, Mohammed VI University Hospital, Epidemiological Laboratory of Clinical Research and Public Health, Oujda, MAR; 2 Cardiology, Mohammed I University, Mohammed VI University Hospital, Epidemiological Laboratory of Clinical Research and Public Health, Oujda, MAR; 3 Cardiology, Mohammed I University, Mohammed VI University Hospital, Oujda, MAR

**Keywords:** cancer-associated thrombosis, acute myocardial infarction, pulmonary embolism

## Abstract

Patients with cancer are at major risk for both venous and arterial thrombotic complications. Venous involvement of cancer-associated thrombosis encompasses deep vein thrombosis and pulmonary embolism. Arterial manifestations include mainly stroke and myocardial infarction. We present the case of a 59-year-old woman admitted to the hospital for chest pain of five hours duration. She had been diagnosed with advanced lung cancer one month before. Electrocardiogram showed ST-segment elevation in all leads except aVR, suggesting a myocardial infarction. Coronary angiography revealed thrombi in both the right coronary artery and the left anterior descending coronary artery in the absence of any atherosclerotic lesions. Tirofiban infusion was administered; furthermore, a computed tomographic pulmonary angiography showed a distal pulmonary embolism. The patient progressed well and was discharged on anticoagulation with vitamin K antagonist. These findings highly imply that the malignancy altered the patient’s blood coagulability and induced the formation of the thrombi ensuing acute myocardial infarction and pulmonary embolism. We will emphasize the relationship between cancer and thrombosis with a special focus on the conservative management strategy with anticoagulant and antiplatelet therapy in acute coronary syndrome without evidence of atherosclerotic lesions.

## Introduction

Thromboembolism, including venous and arterial events, is among the causes of death in cancer patients [1]. The relationship between cancer and thrombosis has been amply studied over the years [2]. Nevertheless, there is very limited information regarding arterial thromboembolism in malignancy. We herein present a rare case of intracoronary thrombosis associated with pulmonary embolism successfully treated by means of glycoprotein IIb/IIIa inhibitor infusion and acenocoumarol in a woman with advanced broncho-pulmonary adenocarcinoma and discuss the potential underlying mechanism.

## Case presentation

**Figure 1:**
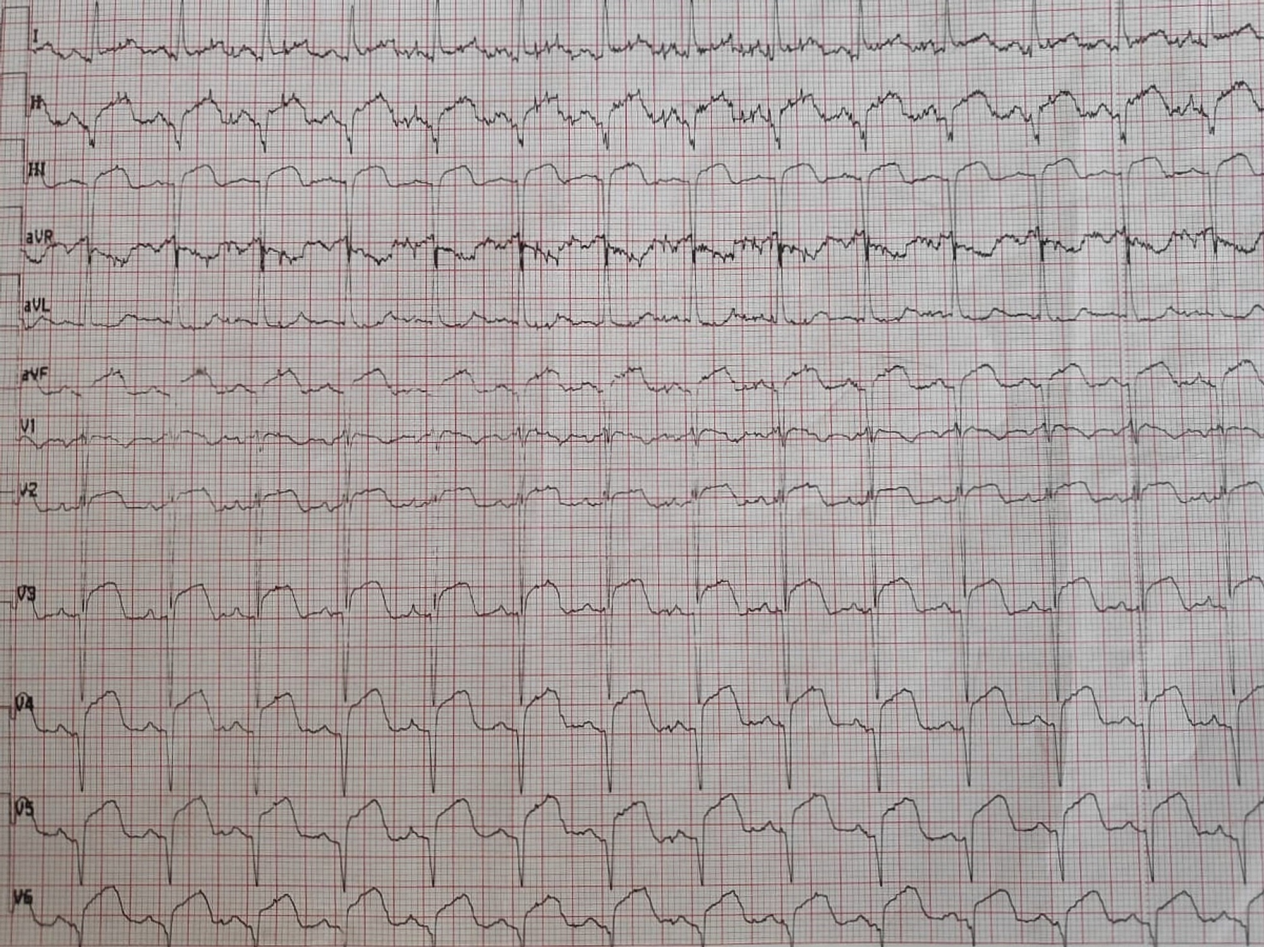
Electrocardiogram showing ST segment elevation in all leads, except aVR where ST segment depression was noted

**Figure 2:**
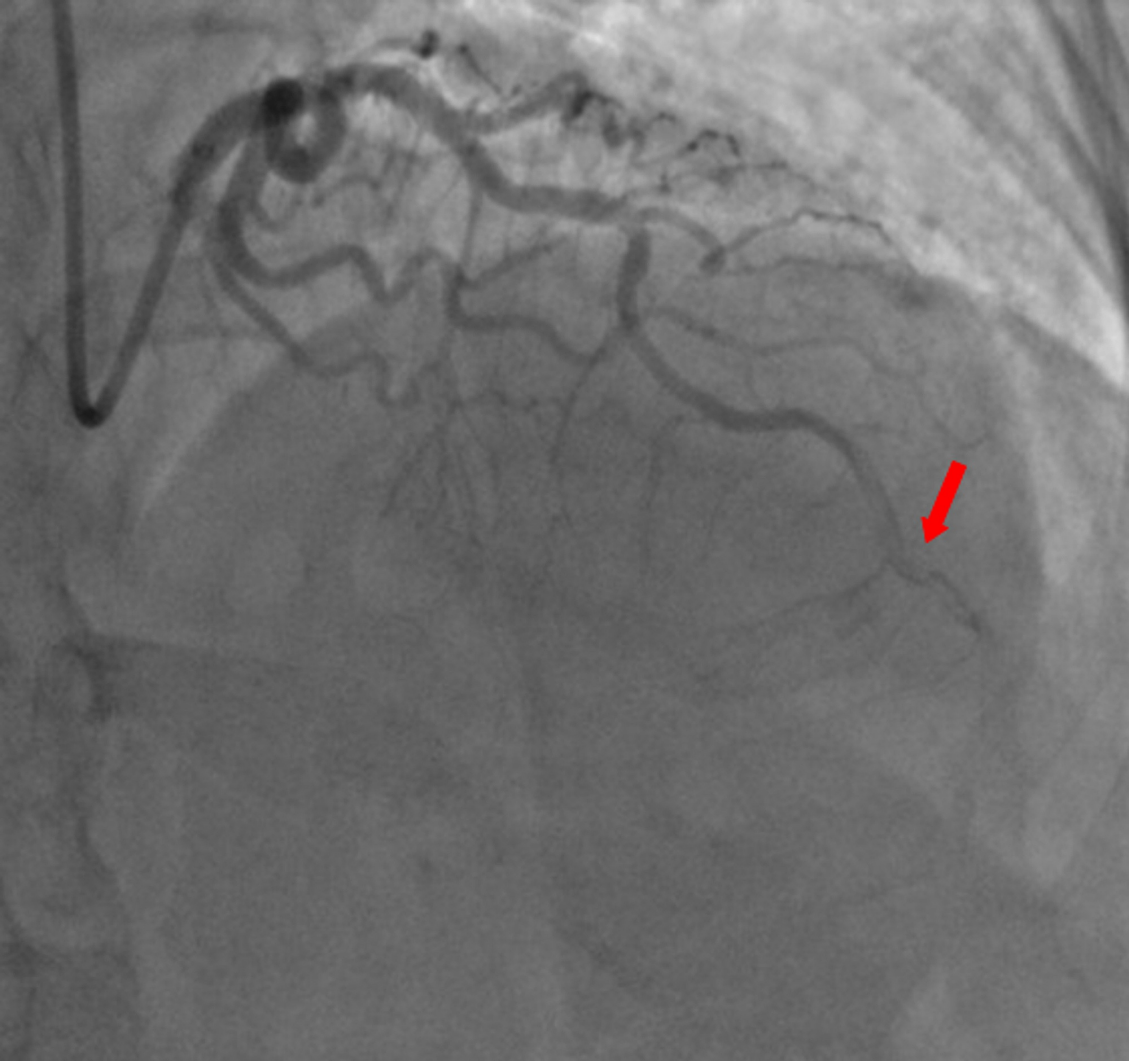
Coronary angiography showing an extended thrombus in the distal segment of the left anterior descending artery (LAD)

**Figure 3:**
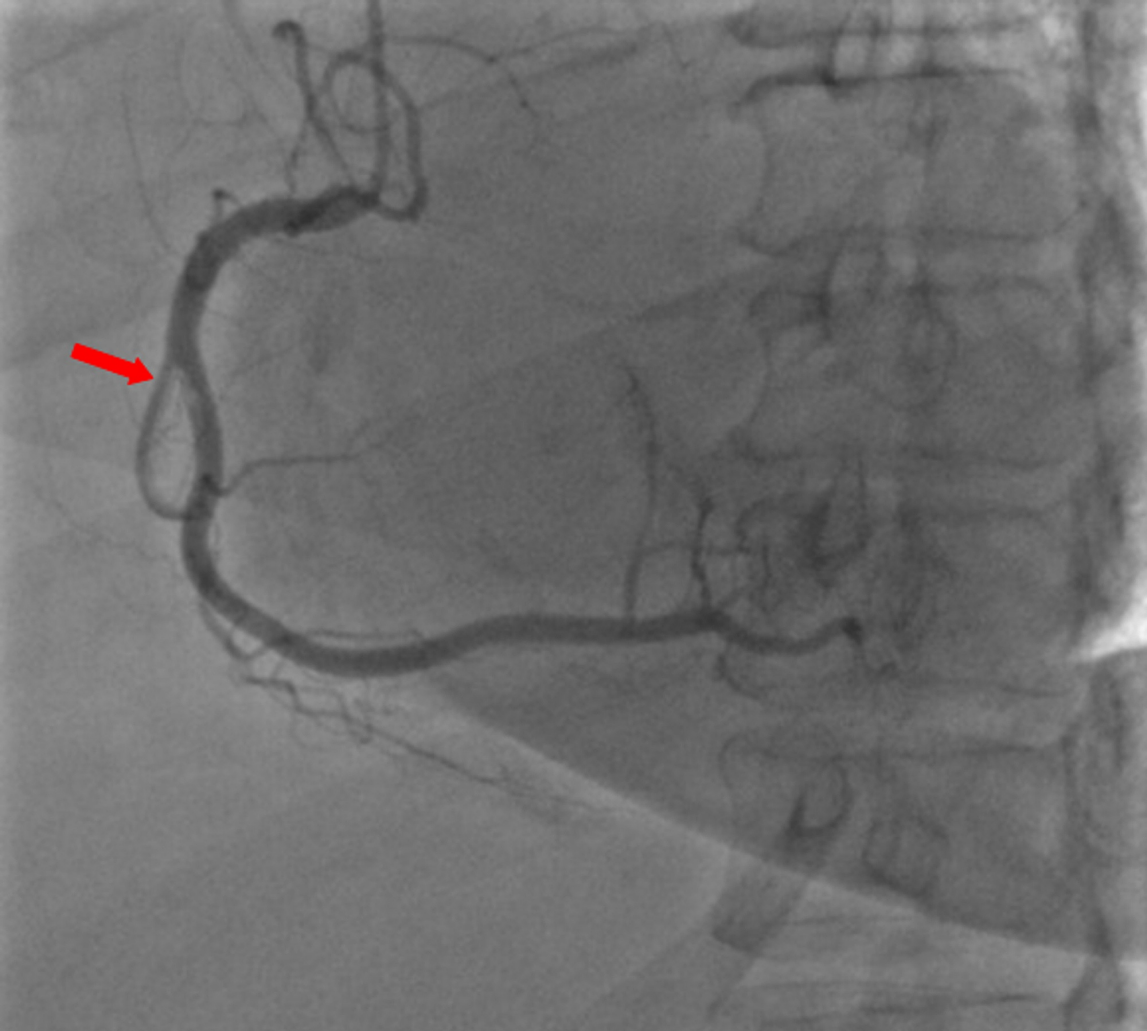
Coronary angiography showing a thrombus in the second segment of the right coronary artery (RCA)

**Figure 4:**
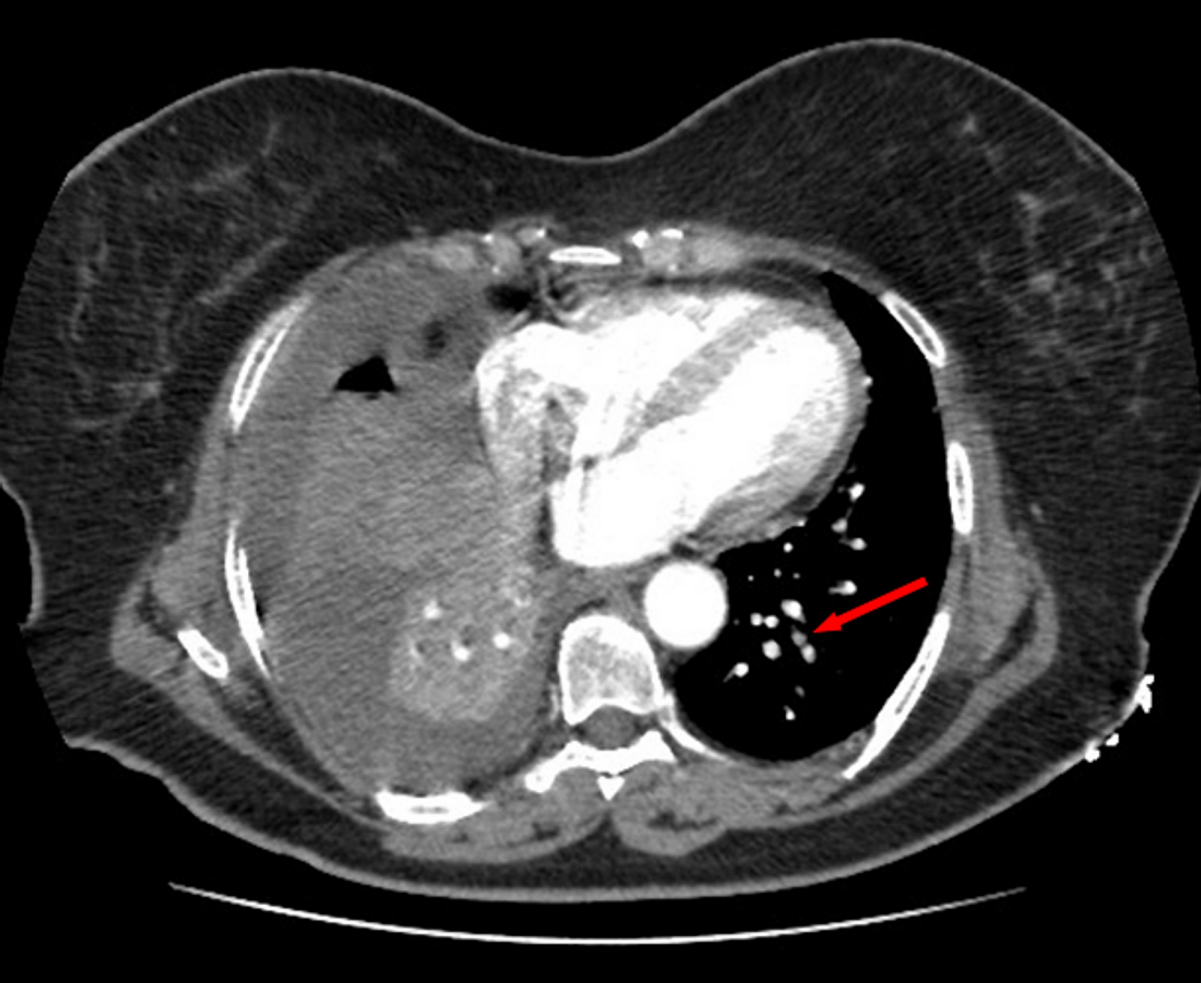
Computed tomographic pulmonary angiography showing a distal pulmonary embolism

A 59-year-old woman was admitted to the emergency department with sudden onset of chest pain for five hours. She had hypertension (treated with β-blockers) as a cardiovascular risk factor. She was diagnosed with advanced non-small-cell lung cancer with liver metastases one month previously. The patient was not considered for surgical resection, she was scheduled for chemotherapy, and, consequently, no antineoplastic treatment had been initiated yet. Prophylactic anticoagulation was not indicated On clinical examination, her blood pressure was 150/90 mmHg with a pulse of 100 beats per minute, oxygen saturation of 95%, and normal heart sounds. The 12-lead electrocardiogram showed sinus rhythm with a heart rate of 102 beats for minutes and ST-segment elevation in all leads except aVR where ST-segment depression was noted (Figure 1). Her laboratory test results revealed a baseline troponin T level of 1212 ng/ml (normal range < 26 ng/ml). Also, the echocardiography findings revealed akinetic walls from the mid to apical septum and anterior and inferior walls. The ejection fraction was estimated to be 39%. Therapy was started with clopidogrel, aspirin, and low-molecular-weight heparin. The patient underwent a coronary angiogram from the right radial approach, which revealed the presence of an extended thrombus in the left anterior descending artery (LAD), and another thrombus was found in the second segment of the right coronary artery (RCA) with thrombolysis in myocardial ischemia (TIMI) III flow and without any atherosclerotic lesions in the coronary artery tree (Figure 2 and Figure 3), therefore, balloon angioplasty and/or stent placement was not considered. The patient was taken to the coronary care unit (CCU) and tirofiban infusion was administered for a period of 48 hours. In view of persistent breathlessness, pulmonary embolism was suspected. There were no symptoms or clinical signs suggestive of deep venous thrombosis. A computed tomographic pulmonary angiography was performed and showed a distal pulmonary embolism (Figure 4). The patient was discharged on acenocoumarol to be followed as an outpatient. At her 45 days follow-up, she did not report any episode of chest pain, bleeding, as well as any thrombotic events.

## Discussion

Malignancy is a recognized risk factor for deep vein thrombosis, pulmonary embolism, and even arterial thromboembolism, including peripheral thrombosis, stroke, and myocardial infarction [3]. Thrombosis represents the second most common cause of death in cancer patients [4]. Several determinants are considered to participate in hypercoagulability in malignancy. The secretion of pro-inflammatory cytokines and pro-coagulants from neoplastic cells causes endothelial damage in many vascular territories. The consequence is upraised vascular permeability for platelet-activating factors and tissue factors, decreased inhibitors of coag­ulation, and impaired fibrinolysis, which increase the risk of thrombosis [5-6]. The present case describes a rare case of intracoronary thrombosis in a 59-year-old woman recently diagnosed with lung cancer. The coronary angiography had not revealed any significant coronary stenosis in the entire coronary artery tree. It is reasonable that the hypercoagulable state of cancer was responsible for intracoronary thrombosis. This is consistent with a few previously reported cases.

To the best of our knowledge, acute myocardial infarction consequential to hypercoagulability induced by malignancy is a rare incident. The majority of arterial thrombosis in cancer patients is situated in the lower extremities, especially the femoral arterial bed. Coronary arteries are not the usual area of thrombosis in cancer patients. The relationship between the type of cancer and thrombosis is conflicting. An analysis conducted by Oren and Herrmann showed that cancer types highly accompanied with an increased risk of arterial thromboembolism encompassed lung [hazard ratio (HR) 9.6], pancreas (HR 6.8), colorectal (HR 6.7), and gastric cancer (HR 6.0) [7]. Additional data analysis indicated a greater prevalence of arterial thromboembolic incidents in the first six months from the time of cancer diagnosis [8]. In this case, the patient had been diagnosed with lung cancer one month before the event. Likewise, it was demonstrated that advanced cancers are associated with a high incidence of thromboembolism events [8].

No well-defined data exist on arterial thrombosis secondary to cancer. Therefore, treating cancer-induced coronary thrombosis remains challenging. Previous studies reported that anticoagulation and antiplatelet therapy is key in acute coronary thrombosis without any underlying atherosclerotic plaque [7]. In our case, no stent implantation, balloon dilatation, or thromboaspiration were performed. This approach was adopted for different reasons: the absence of coronary artery atherosclerotic plaque responsible for coronary flow impairment and to avoid the risk of further distal embolization. A conservative strategy with anticoagulant and antiplatelet therapy was adopted; anticoagulation with vitamin K antagonists (VKA) was believed to be adequate owing to the concomitant occurrence of pulmonary embolism and acute coronary thrombosis.

The decision to use anticoagulants and antiplatelets or not must be founded on a risk-benefit evaluation of bleeding versus a recurrent thromboembolic event. Low-molecular-weight heparin (LMWH) is recommended as a first-line treatment for cancer-associated thrombosis. Direct oral anticoagulants (DOACs) have recently emerged as a new therapeutic option [9]. Despite these clear guidelines, oral VKA are overused in our context of middle-income countries, given the relatively low cost and oral route instead of subcutaneous LMWH administration, which is not supported in patients with terminal cancer and may reduce patient adherence. The presented case revealed that a conservative management strategy with anticoagulant and antiplatelet therapy may be well-tolerated and effective in cancer patients with acute coronary syndrome unaccompanied by evidence of coronary artery stenosis.

## Conclusions

We report an unusual case of thrombosis in two different vascular territories: in the lung (pulmonary embolism) and the heart (acute myocardial infarction with thrombi in two different coronary arteries). This case highlights the prothrombotic state of malignancies. Future studies must attempt to analyze the optimal strategies to treat and prevent arterial thromboembolism in patients with cancer.
